# Unpacking PCOS Inflammation: From Misconceptions to Immune Networks

**DOI:** 10.1210/endocr/bqaf166

**Published:** 2025-11-06

**Authors:** Gabriela De Robles, Kiara D Wiggins, Zena Del Mundo, Naveena Ujagar, Christy M Nguyen, Marcus M Seldin, Gabriela Pacheco-Sanchez, Dequina A Nicholas

**Affiliations:** Department of Molecular Biology & Biochemistry, Charlie Dunlop School of Biological Sciences, University of California Irvine, Irvine, CA 92697, USA; Department of Biological Chemistry, School of Medicine, University of California Irvine, Irvine, CA 92697, USA; Department of Molecular Biology & Biochemistry, Charlie Dunlop School of Biological Sciences, University of California Irvine, Irvine, CA 92697, USA; Department of Molecular Biology & Biochemistry, Charlie Dunlop School of Biological Sciences, University of California Irvine, Irvine, CA 92697, USA; Department of Molecular Biology & Biochemistry, Charlie Dunlop School of Biological Sciences, University of California Irvine, Irvine, CA 92697, USA; Department of Molecular Biology & Biochemistry, Charlie Dunlop School of Biological Sciences, University of California Irvine, Irvine, CA 92697, USA; Department of Biological Chemistry, School of Medicine, University of California Irvine, Irvine, CA 92697, USA; Department of Biological Chemistry, School of Medicine, University of California Irvine, Irvine, CA 92697, USA; Department of Molecular Biology & Biochemistry, Charlie Dunlop School of Biological Sciences, University of California Irvine, Irvine, CA 92697, USA; Department of Molecular Biology & Biochemistry, Charlie Dunlop School of Biological Sciences, University of California Irvine, Irvine, CA 92697, USA; Department of Biological Chemistry, School of Medicine, University of California Irvine, Irvine, CA 92697, USA

**Keywords:** cell-to-cell communication, inflammation, PCOS, tissue, systemic, reproductive immunology

## Abstract

Polycystic ovary syndrome (PCOS) is a complex endocrine disorder affecting women worldwide. For decades, the “chronic inflammation hypothesis” has guided research into the role of the immune system in PCOS pathogenesis. However, this review challenges this paradigm by pointing out discrepancies in current literature on systemic immune markers in PCOS. We highlight the limitations of relying solely on systemic inflammatory markers and emphasize the importance and diversity of tissue-specific immune responses. Evidence from human and animal studies reveals distinct immune responses across various tissues affected by PCOS or inflammation, including the hypothalamus, pituitary, ovaries, endometrium, and adipose tissue. These findings suggest that PCOS is not characterized by systemic low-grade inflammation, but rather by discrete tissue-specific immune interaction with endocrine cells. Finally, we discuss how advanced single-cell technologies and computational tools are enhancing our understanding of immune cell signaling to endocrine cells in PCOS. Moving forward, we propose that research should focus on elucidating causal relationships between local immune responses and endocrine dysfunction in PCOS. This shift in perspective from systemic to tissue-specific immune responses is critical for developing targeted immunotherapies for PCOS.

For almost 3 decades, scientists have been trying to understand the relationship of inflammatory markers to polycystic ovary syndrome (PCOS), the most common endocrine disorder in women worldwide. Elevated C-reactive peptide (CRP, an inflammatory marker) observed in women with PCOS led to the “chronic inflammation hypothesis.” However, systemic inflammatory markers that would support this hypothesis of low-grade chronic inflammation are not generally elevated in women with PCOS. In this review, we revisit the “chronic inflammation hypothesis” and survey the current literature that supports a need to evaluate the role of the immune system in PCOS. We highlight the limitations and misconceptions generated from literature focused on analysis of systemic markers and make the case that to understand how the immune system is linked to PCOS, the field must take tissue-specific approaches. Importantly, we demonstrate that exploration of immune-endocrine communication networks within tissues is the future of deconvoluting the role of the immune system in PCOS. Finally, we consolidate resources to support a shift in perspective of clinicians and researchers as they study immune dysregulation in PCOS.

## Systemic Immune Markers in PCOS

The widely accepted concept of chronic inflammation in PCOS has evolved through a series of key studies analyzing systemic immune markers (Table 1). Gonzalez et al made the first observation of the elevated inflammatory marker tumor necrosis factor (TNF)-α in normal weight women with PCOS (1). Ridker et al followed, reporting an association of cardiovascular disease risk with CRP (2, 3), an acute phase protein used as a marker of low-grade chronic inflammation. Kelly et al demonstrated that CRP was elevated in women with PCOS and proposed the “chronic inflammation hypothesis” (4). This study popularized the idea that chronic inflammation, inferred through measurement of CRP, is a core feature of PCOS. Despite cautions against the “chronic inflammation hypothesis” due to inconsistent support from literature and several reviews and meta-analyses showing no elevation of key cytokines in PCOS (5) (Table 1), elevated CRP was readily accepted as the clinical inflammatory marker to define chronic inflammation in PCOS (6, 7). The “chronic inflammation hypothesis” currently shapes the field's approach to dissecting how the immune system contributes to PCOS. Therefore, characterization of inflammatory markers in PCOS has been focused on clinical studies evaluating circulating cytokines or CRP in serum or plasma. These studies range from small cohorts well controlled for confounding variables to larger cohorts with women admitted using the Rotterdam criteria, introducing significant variability (6, 8). Individual studies may measure one cytokine or marker of inflammation, while others measure a select few (9, 10). Overall, these approaches have led to a fragmented inconsistent literature on the systemic levels of key cytokines that would confirm the presence of chronic low-grade inflammation including interleukin (IL)-6, TNF-α, and IL-1β. For example, Tosatti et al found TNF-α levels to be lower in women with PCOS compared to healthy controls (11). Further, a few studies found no significant difference in circulating levels of IL-6 and TNF-α (11, 12), while one study reported that women with PCOS had extremely elevated levels of IL-6, IL-18, and TNF-α, comparable to those typically seen in cases of infection or injury (13).

**Table 1.** Summary of common systemic inflammatory markers in analyzed in PCOS studies

Marker	Change in PCOS	Study population	References
CRP	Elevated	BMI-matched women with PCOS defined by androgen excess and oligo-ovulation	Kelly et al (2001) (4)
	Significantly elevated	Meta-analysis of 63 studies	Aboeldalyl et al (2021) (6)
TNF-α	Elevated	BMI-matched normal weight women with PCOS	Gonzalez et al (1999) (1)
	No difference	BMI-matched obese women with PCOS	Gonzalez et al (1999) (1)
	Lower	BMI-matched women with PCOS	Tosatti et al (2020) (11)
	No difference	BMI-matched women with PCOS	Ciaraldi et al (2013) (12)
	Extremely elevated	Women with PCOS (comparable to infection/injury levels)	Al-Musawy et al (2018) (13)
	No difference	Meta-analysis of 9 studies	Aboeldalyl et al (2021) (6)
IL-6	No difference	BMI-matched women with PCOS	Tosatti et al (2020) (12); Ciaraldi et al (2013) (12)
	Extremely elevated	Women with PCOS (comparable to infection/injury levels)	Al-Musawy et al (2018) (13)
	Increased	Meta-analysis of 9 studies	Aboeldalyl et al (2021) (6)
	Increased	Meta-analysis of 4 studies (high variability)	Bansal et al (2025) (14)
IL-1β	No difference	Women with PCOS vs controls	Ciaraldi et al (2013) (12)
IL-18	Extremely elevated	Women with PCOS (comparable to infection/injury levels)	Al-Musawy et al (2018) (13)
IFN-γ	No difference	BMI-matched women with PCOS	Tosatti et al (2020) (11)
	No difference	Women with PCOS vs controls	Ciaraldi et al (2013) (12)

Abbreviations: BMI, body mass index; CRP, C-reactive protein; IFN-γ, interferon-γ; ; IL-, interleukin; PCOS, polycystic ovarian syndrome; TNF-α, tumor necrosis factor alpha.

Reviews and meta-analyses have helped consolidate this literature (6, 9), although unified conclusions regarding changing in circulating immune factors remain elusive. Abodoelyl et al found that CRP is significantly elevated in a meta-analysis of 63 studies. In a subset of 9 studies that also measure IL-6 and TNF-α, IL-6 was increased while no difference was observed for TNF-α (6). A separate meta-analysis reported increased IL-6 but only analyzed 4 papers and also reported extremely high variability along with a publication bias (14). This current meta-analysis consolidates and organizes the breadth of cytokines measured across PCOS studies and is a comprehensive resource. Due to the detection of elevated cytokines only in select experimental conditions, the current clinical literature does not provide convincing evidence that systemic chronic low-grade inflammation is occurring in PCOS as indicated by serum cytokines. This is in clear contrast to the cardiovascular literature, where elevated CRP is accompanied by increased levels of pro-inflammatory cytokines, such as IL-1, IL-6, interferon (IFN)-γ, and TNF-α (15-17).

Despite the lack of consensus on cytokines in PCOS (9, 18), it is clear that CRP is elevated in disease although the cause of this elevation is unknown. CRP is an imperfect stand-alone marker of chronic inflammation. CRP is an acute phase inflammatory protein primarily produced by the liver in response to cytokines IL-6 and IL-1β. However, multiple cellular sources can secrete CRP; and further, monomeric vs pentameric forms of CRP have opposing immune functions and are not discriminated against by the common hsCRP clinical test (19). Further, in PCOS, CRP does not correlate with androgens and elevation of CRP in women with PCOS is not significant when data is controlled for insulin sensitivity (4), casting additional doubt on low-grade chronic inflammation as an important factor in disease pathogenesis. Although the role of chronic inflammation in PCOS is unclear, elevated CRP indicates that the immune system is important to disease etiology.

In summary, despite the strides in clinically identifying elevated CRP in PCOS patients, there is a lack of evidence establishing the causal relationship between the immune system and PCOS. Serum cytokine profiling is inconsistent across studies and patient populations. Moreover, the interpretation of systemic inflammatory markers is limited because their source and target tissues remain unknown. Though serum is easily accessible, analysis of immune cells, and more importantly tissues, is necessary to deconvolute the immune mechanisms that contribute to PCOS. Mouse models of PCOS reviewed in (20) provide a platform for assessing tissue-specific immune changes and establish cause and effect using classical immunological approaches (21). Finally, PCOS is uniquely a human disease, and the tools to support creative approaches to tackle the immune system's role in PCOS are emerging.

## Tissue-Specific Immune Function in PCOS

Mounting evidence from analysis of systemic immune markers implicates immune dysregulation as a critical factor in PCOS. However, systemic markers are indicators of immune action within tissue sites, the location where immune cells function (22). Yet, the role of tissue-specific immune action as a potential driving force behind this complex condition remains understudied. Reproductive symptoms of PCOS are driven by environmental and genetic dysregulation of endocrine processes in tissues of the hypothalamic-pituitary-gonadal (HPG) axis which orchestrates reproductive function through tight temporal control of neuropeptides and hormones, including gonadotropin-releasing hormone (GnRH), follicle-stimulating hormone (FSH), luteinizing hormone (LH), and sex steroids such as estrogen, progesterone, and androgens (23). PCOS also has several comorbidities, including metabolic syndrome and cardiovascular risk, implicating many other tissues including adipose and heart. While some data on immune function in tissues from women with PCOS has been generated (Table 2), the scarcity of and lack of access to human tissues have led researchers to rely on rodent models (20). Current animal models (prenatal/postnatal androgen treatment, anti-Mullerian hormone administration, and aromatase inhibition) are hyperandrogenic models which primarily recapitulate the hormonal environment of PCOS but cannot fully capture the genetic and epigenetic factors underlying human PCOS pathogenesis. Despite this limitation, these models serve as tools for mechanistic investigation of tissue-specific immune changes in PCOS produced by androgen excess.

**Table 2.** Summary of studies assessing tissue-specific immune parameters in PCOS

Tissue type	Species	Data type
Hypothalamus	Mouse	Bulk RNA sequencing (75)
	Rat	ELISA, Western blotting (24)
Ovary	Human	scRNA sequencing (69), RT-qPCR (42)
	Mouse	Bulk RNA sequencing (75), Histology, qPCR, chemistry analysis with autoanalyzer (76), scRNA sequencing and spatial transcriptomics (67) Flow cytometry (43), Western blotting, ELISA, Immunohistochemistry (45)
	Rat	scRNA sequencing (68), Histology, ELISA (77)
Ovary (granulosa cells)	Human	RT-qPCR, Western blotting, and ELISA (41), Microarray and scRNA sequencing (78)
	Mouse	scRNA sequencing (79), RT-qPCR (44)
Ovary (follicular fluid)	Human	Flow cytometry and ELISA (80), RT-qPCR (41)
Endometrium	Human	Western blotting, Immunohistochemistry, Immunofluorescence (47), scRNA sequencing (49)
Uterus	Mouse	Flow cytometry (43)
	Rat	Histology, immunofluorescence, ELISA, Western blot (81)
Adipose	Mouse	Bulk RNA sequencing (75), ELISA, Western blotting (46), Flow cytometry (43)
	Rat	RT-PCR (82)
Cardiac Tissue	Human/Mouse	RNA Sequencing, Flow cytometry, Immunofluorescence (57)
Spleen	Mouse	Flow cytometry (43)
Muscle	Rat	RT-qPCR, Western blotting, Radioimmunoassay, Lipid peroxidation (83)
Kidney	Rat	Histology, ELISA (77)
Liver	Mouse	Histology, qPCR, Chemistry analysis with autoanalyzer (76)
Intestines	Mouse	Western blotting, ELISA, Immunohistochemistry (45)

Abbreviations: ELISA, enzyme-linked immunosorbent assay; PCOS, polycystic ovarian syndrome; RT-qPCR, reverse transcription–quantitative polymerase chain reaction; scRNA, single-cell RNA.

### Hypothalamus

The hypothalamus integrates multiple signals to control the pulsatile secretion of GnRH, thereby acting as a central regulator of reproductive hormones. Hypothalamic neurons are susceptible to immune regulation, and disruptions within the hypothalamus can have cascading effects on gonadotropin secretion and consequently ovarian function, ultimately contributing to the anovulation and hyperandrogenism characteristic of PCOS.

A study from letrozole treated rats showed inflammatory cytokines IL-6, IL-1β, and TNF-α are elevated in the hypothalamus (24). Although outcomes from studies are contradictory, these cytokines may impact GnRH neuronal activity, leading to altered LH pulsatility and downstream hormonal imbalance (25). Aside from cytokines, microbial components known to be elevated in PCOS such as lipopolysaccharide (LPS) (26) can induce local immune activity within tissues. For example, intrauterine administration of LPS in ovariectomized female rats reduces the number of Kiss1-expressing neurons in the arcuate nucleus, highlighting how immune signaling in the hypothalamus can directly impair the reproductive axis (27). However, our understanding of immune involvement in the hypothalamus is limited because the hypothalamus does not function in isolation. Immune signals from circulation may trigger or enhance immune responses in the central nervous system. This interconnection raises important questions about how communication between different organs contributes to reproductive disorders.

### Pituitary

Patients with PCOS typically present with excess androgens, a high LH to FSH ratio driven by abnormally elevated LH, and more recently described, reduced prolactin levels (23, 28-31). This dysregulation of pituitary-related hormone secretion warrants the identification of specific mediators. Acute LPS administration in male mice and chronic LPS administration in female mice increases plasma LH and FSH (32, 33). A similar trend is seen in geese at 24 to 36 hours post-LPS delivery (34). Early work in ewes demonstrated that acute bolus of LPS reduces GnRH responsivity of gonadotropes (35, 36). More recent experiments confirm that acute LPS challenge in ewes results in decreased LH 3 hours after injection, while 7 days of daily LPS delivery leads to decreased LH and increased FSH (37). Acute changes in hormones in response to LPS establishes that local immune mechanisms within the pituitary interpret inflammatory cues and influence endocrine cell functions. Further, the species- and time-dependent responses to LPS underscore the complexity of studying the impact of inflammation on pituitary hormone secretion.

Recent single-cell transcriptomic studies in mice reveal intricate interactions between pituitary hormone-producing cells and immune cells. Macrophages and neutrophils infiltrate the pituitary after LPS treatment, altering hormone transcription levels, including those from gonadotropes, LHβ and FSHβ (38). Additionally, pituitary-resident macrophages can modulate hormone secretion, suggesting a link between inflammation and hormone regulation (39). Given the elevation of CRP in women with PCOS, it is likely that changes in immune function within the pituitary contribute to elevated LH in PCOS. Therefore, complex feedback mechanisms within the HPG axis due to varying hormone targets, studying the impact of immune changes at the tissue level is essential for a comprehensive understanding of PCOS.

### Gonads (Ovaries)

The ovary is not only an important site of sex hormone production and reproductive function in PCOS, but it is also a site of localized immune-endocrine interaction. Human studies demonstrate elevated levels of pro-inflammatory cytokines such as IL-6, IL-1β, and IL-18 in the follicular fluid of individuals with PCOS. In vitro, these cytokines activate inflammatory signaling in a granulosa cell line including nuclear factor κB (NFκB) and inflammasomes, resulting in oxidative stress and impaired proliferation (40, 41). Interestingly, Lai et al demonstrated that elevated follicular fluid fatty acids correlate with cytokines and induce cytokine transcripts in vitro in a granulosa cell line through ERK1/2 and inflammasome activation (42). These data provide a link between systemic metabolism and local tissue immune responses. Further investigations into the ovarian immune landscape using mice have revealed additional insights. Flow cytometry analysis showed an overall increase in CD4+ T helper cells in ovaries of dihydrotestosterone (DHT)-exposed mice (43). These outcomes implicate local ovarian inflammation in the disruption of normal folliculogenesis and development of cysts.

Systemic immune signals also shape the ovarian microenvironment. Peripheral immune cells can enter the ovary and amplify local inflammatory signals through cytokine secretion, although no exact mechanisms have been defined (41). Microbial products such as LPS can also have effects on the ovary. The natural ligand for toll-like receptor 4 (TLR4) is LPS. Granulosa cells express TLR4 and respond to LPS stimulation with inflammatory gene expression and a reduction of estrogen likely due to inhibition of Cyp19 (44). Together, these data suggest that local cytokine action and the well-characterized TLR4–MyD88–NFκB pathway initiates a feed-forward loop that impairs oocyte quality and hormone productions.

Several immune interventions support the importance of immune-endocrine crosstalk in the ovaries for PCOS pathogenesis. Recent work in a dehydroepiandrosterone (DHEA) mouse model of PCOS demonstrated that curcumin, a polyphenolic lipophilic compound with anti-inflammatory properties, improves reproductive outcomes (45). Concurrent with reproductive improvement was reduced ovarian pro-inflammatory cytokines IL-6, TNF-α and IL-17 and TLR4/MyD88/NF-κB pathway activation. Lang et al demonstrated that treatment of letrozole-induced PCOS mice with etanercept (ETA), an inhibitor of TNF-α and TNF-β, reduced weight gain, testosterone levels, and improved follicle development (46). The few studies analyzing the local ovary immune environment in PCOS have been fruitful, and this area is ripe for development of targeted immunotherapies to improve outcomes.

### Non-HPG Axis Tissues

Tissue-specific immune dysfunction extends beyond the HPG axis, affecting various tissues, including endometrium, adipose, and heart. The tissue-specific immune environments in PCOS may play a role in the comorbidities associated with the condition. This includes miscarriage, metabolic syndrome, insulin resistance, and even risk for cardiometabolic disease.

In PCOS patients, the endometrium exhibits significant increases in inflammatory cytokines and signaling pathway activation. Endometrial biopsies from PCOS patients revealed increased expression of TLR4 and several downstream mediators of TLR4 signaling including NFκB (47). TNF-α was found to be elevated in the endometrium of women with PCOS. This elevation correlated with increased NFκB and reduced GLUT4 expression, a glucose transporter important for tissue cell health. In vitro treatment of endometrial samples from women with PCOS revealed that TNF-α can directly suppress endometrial GLUT4 expression, likely via NFκB activation (48). Furthermore, a population of endometrial epithelial cells from PCOS patients shows upregulation of the long-noncoding RNA NEAT1 (49), which activates inflammasomes and is associated with implantation failure (50, 51), suggesting that specific cell populations may be particularly affected by PCOS-related inflammatory processes. In vitro, metformin, known to have anti-inflammatory actions, reduces TNF-α expression in endometrial samples from women with PCOS. Inhibition of local endometrial inflammation is a potential mechanism that mediates the known effects of metformin on reducing miscarriage risk in women with PCOS (47, 52). Together, these data suggest direct cytokine action as potential mechanisms contributing to endometrial dysfunction and miscarriage and highlight potential therapeutic targets.

Adipose tissue, an active endocrine organ, secretes adipokines that influence the HPG axis while being directly affected by pituitary hormones such as growth hormone, FSH, and TSH. In the prenatal testosterone sheep model of PCOS, subcutaneous and visceral fat depots show increased inflammatory cytokine and macrophage markers (53). Sequencing of these depots reveal a mixed transcriptomic profile with no clear patterns that define inflammation. The differentially expressed genes in adipose of prenatal testosterone sheep model include an increase in the inhibitor of NFκB and an increase in AP-1, a potent transcriptional co-activator (54). Furthermore, the traditional Chinese medicine Qi Gong Wan prescribed for obese women with infertility downregulates adipose gene expression of TNF-α while reducing adipocyte hypertrophy in the letrozole and high-fat diet-induced PCOS mouse model (55). In agreement, Lan et al also demonstrated that blocking TNF-α using etanercept decreased the size of adipocytes in a letrozole-induced rat PCOS model (46). Reduction in adipocyte size is linked to improved insulin sensitivity and fasting plasma insulin (56), indicating that targeting adipose inflammation may improve metabolic outcomes in PCOS. Women suffering from PCOS face increased prevalence of coronary artery disease in addition to their high risk of poor metabolic outcomes. Cardiac tissue from PCOS patients displaying increased macrophage accumulation as measured by transcriptomic profiling and immunostaining (57). These immune changes in the hearts of PCOS patients represent a new frontier to explore that may have benefits for cardiovascular health.

### Limitations to Extrapolating Tissue-Specific PCOS Immune Changes From Animal Models to Humans

While animal models have provided valuable insights into tissue-specific immune alterations in PCOS, several limitations must be acknowledged when extrapolating findings to human PCOS. First, PCOS is a complex disorder with a pathogenesis that is determined by genetic and epigenetic factors, which in turn underlie hyperandrogenism (58-62). Current animal models can only reproduce tissue-specific immune alterations resulting from androgen excess, not those that are genetically or epigenetically determined. For these reasons, the relationship between inflammation and genetic or epigenetic mechanisms remains unclear. An important consideration is that ovaries are not the only site of hyperandrogenism in PCOS. For example, it is estimated that over 50% of women with PCOS suffer from adrenal hyperandrogenism (63), while adipocytes from women with PCOS have been demonstrated to be intrinsically hyperandrogenic (64). It is unknown if current animal models replicate these unique pathophysiological features, and therefore we do not know the impact of these androgen sources on tissue-specific immune changes in PCOS. The intrinsic hyperandrogenism of adipocytes and the adrenal gland may contribute to tissue-specific immune alterations independent of systemic androgen levels. Whether tissue-specific immune alterations are genetically determined, occur downstream of elevated hormones, or both, cannot be fully addressed by current animal models. Finally, the contribution of hormones other than androgens to immune microenvironments may not recapitulate the human condition, especially in aromatase inhibition models that may reduce systemic estrogen, a known modulator of immune function.

In summary, the HPG axis and associated tissues are immunologically active sites whose dysfunction and inflammatory status contributes to PCOS. Although human tissue samples are optimal for maximizing the translational potential of data generated to understand the contribution of immune function to the etiology and pathophysiology of PCOS, access is limited and many tissue types have not been investigated due to ethical consideration. However, the literature on tissue-specific immune status in PCOS is growing, with efforts like the recent publication by Torstensson et al leading the way (43). They provide clear demonstrations that androgens have tissue-specific effects in mice that cannot be captured in blood, such as increased uterine IL-18, which agree with human data. This study, though in mice, provides further support that PCOS is not a disease of chronic systemic inflammation, but rather of tissue-specific immune dysregulation. A summary table has been compiled to present tissues that have been investigated and shown to exhibit inflammatory changes in either PCOS patients or animal models (Table 2). It is worth noting that the pituitary gland is not included in this table, as the role of the pituitary immune network in the context of PCOS has not yet been characterized. Investigating the communication between immune and endocrine cells within these tissues is necessary to understand PCOS's complex pathophysiology and develop targeted immunotherapy for PCOS.

## Immune-Endocrine Crosstalk in PCOS

Recent advancements in single-cell analysis, including flow cytometry, imaging, and sequencing, have enhanced our ability to analyze cellular environments and determine how immune and endocrine cells communicate in PCOS. Few single-cell studies in PCOS exist and for those that do, most focus on ovarian and endometrial tissues and characterizing changes in immune populations. Despite this dearth of literature, lessons can be learned from both single-cell studies investigating immune changes in the female reproductive tract in relevant physiological conditions and from PCOS models. Single-cell studies at the tissue level are moving our understanding of immune dysregulation in PCOS from characterization toward signaling networks and ultimately to cause and effect.

### Immune-Endocrine Cell Communication in Rodent Reproductive Tissues

A comprehensive single-cell sequencing study of the mouse reproductive tract throughout the estrous cycle revealed valuable insights into the reproductive immune compartment, providing a foundation for investigating its role in PCOS (65). Within the ovary, immune populations do not change with estrous stage, in contrast to cyclical inflammation in the lower reproductive tract, consistent with the tissue remodeling (65, 66). A single-cell atlas of the aging mouse ovary confirmed these results. However, focused analysis on the immune compartment revealed an accumulation in CD4+ T cells in the mouse ovary with age when fertility significantly declines (66). These changes were confirmed by flow cytometry and mirror the increase in CD4+ T cells observed in the ovaries of the DHT mouse model of PCOS (43).

Moving past characterization, several studies have leveraged single-cell datasets to identify potential changes in cell-to-cell communication within the ovary by cycle stage, with age, and in PCOS. IFN-γ signaling to fibroblast was identified as a communication mode specific to the ovary in contrast to the rest of the reproductive tract (65). With age, authors found a correlation of increasing TGF-β with ovarian fibrosis, implicating macrophages signaling to theca cells and fibroblasts (66). In a dehydroepiandrosterone (DHEA) mouse model of PCOS, KEGG pathway analysis of single-cell ovarian transcripts identified cytokine to cytokine receptor interaction as the top pathway represented in PCOS compared to controls. These pathways included IL-17, TGF-β, TNF, and IFN signaling in granulosa cells (67). Despite the clear evidence of immune and endocrine crosstalk, network analysis was only presented for granulosa cell to theca cell communication. A separate study using the letrozole-induced PCOS rat model, did comprehensive analysis on communication networks within the ovary (68). Similar to other studies, they identified macrophages as an important immune cell source of signaling mediators that could initiate crosstalk with theca cells (67, 68). Moreover, natural killer cells were found to be most likely to communicate with other cells in the ovary in PCOS. These data agree with flow cytometry analysis in the DHT model of PCOS demonstrating an increase in CD69+ (activated) NK cells in mice ovary (43). As information on cell-to-cell communication networks in PCOS emerges, it is important to consider spatial localization because communication networks are impacted by proximity.

### Immune-Endocrine Cell Communication in Human Reproductive Tissues

While samples from human PCOS patients have been limited, analyses have been performed on ovaries and endometrium to investigate ligand-receptor interactions. Single-cell RNA sequence data from human adult ovaries was used to deconvolute bulk RNA sequencing data from ovaries of women with PCOS. This computational approach identified an increase in T cells in the ovary in PCOS which aligns with a study in mice (43). In contrast to a mouse study, T cells, rather than macrophages, were identified as the cell type with the most communication with theca cells (67-69). Single nuclei RNA-seq data of endometrium from women with PCOS did not detect significant changes in immune cell populations but did identify natural killer cells and macrophages to be the immune cell types with the most differentially expressed genes (49). Increased signaling between macrophage subtypes was identified, while genes involved in collagen pathways important for extracellular matrix turnover were overall downregulated across immune subclusters (49). This loss of gene expression that supports tissue remodeling may contribute to high risk of miscarriage in women with PCOS and is ripe to explore for therapeutic targets.

Single-cell technologies have significantly enhanced the ability to profile cell-cell communication in PCOS. Across mouse models and human tissue, it is evident that local ovarian inflammation and immune crosstalk to granulosa and theca cells are important in disease. Despite the impact of PCOS on immune status varying by tissue, the types of cytokines produced (TNF-α and IFN-γ), immune cells identified (T cells and macrophages), and crosstalk analyzed are relatively consistent across human studies and PCOS rodent models (43, 67, 68). Robust single-cell datasets, both dissociated tissue and intact, must be produced from tissues across the HPG axis in PCOS conditions for our understanding of immune-endocrine crosstalk to move forward.

### The Future of Immune-Endocrine Cell Communication in PCOS

Computational tools to decipher tissue communication networks in PCOS have significantly increased the utility of single cells sequencing datasets. Commonly used tools such as those in Table 3 usually rely on the co-expression of ligand-receptor pairs, which make them powerful predictors of cell communication. However, this strength also limits the ability to distinguish between direct regulatory interactions and indirect responses. Further, most current studies evaluating immune-to-endocrine cell communication remain correlational, lacking mechanistic insight into the directionality of immune-endocrine signaling. Hence, integrating causal inference methods with cell-cell communication analyses is critical to address this limitation. For instance, CausalCell (70) reconstructs directed gene-gene and cell-cell networks from single-cell data using constraint-based algorithms. CausalCCC builds on this approach by integrating ligand-receptor interactions with causal gene networks across sender and receiver cell types, mapping intercellular signaling pathways (71). FlowSig further models directional intercellular signaling across both single-cell and spatial transcriptomics data (72). These frameworks have not yet been widely applied to PCOS; however, they hold strong potential for modeling causal immune signaling, inter-tissue signaling, and endocrine feedback. Developing an integrated framework that combines cell-cell communication with causal inference represents a critical next step toward identifying mechanistic drivers of immune-endocrine dysregulation in PCOS. Moreover, pairing these frameworks with experimental validation through platforms such as transgenic mouse models, human organoids, human induced pluripotent stem cell (iPSC) models (73), or organ-on-a-chip systems (74) will be necessary to move beyond correlation-based inference and toward causal-based understanding of PCOS pathogenesis. In particular, organoid models represent a promising bridge between in silico predictions and in vivo validation. Organoids can recapitulate tissue-specific immune-endocrine interactions in a controlled environment, allowing for mechanistic validation of computational predictions while maintaining physiological relevance. Future organoid models incorporating both immune and endocrine cell types from PCOS-relevant tissues could provide platforms for testing therapeutic interventions targeting specific cell communication networks identified through computational analysis.

**Table 3.** Computational tools to support single-cell and cell communication analysis

Tool name	Analysis type	Input	Platform
Has been applied to datasets in studies focused on PCOS and reproductive health			
CellChat (84)	Cell-cell communication	scRNA-seq	R, Web interface
CellPhoneDB (85, 86)	Cell-cell communication	scRNA-seq	Python, Web interface
NicheNet (87)	Cell-cell communication	scRNA-seq	R
CIBERSORT (88)	Cell subset estimation	Microarray, bulk RNA-seq	R
Monocle 3 (89)	Psuedotemporal trajectory	scRNA-seq	R
Recommended for future studies in PCOS			
CausalCell (70)	Causal inference	scRNA-seq	Web interface
CausalCCC (71)	Causal inference	scRNA-seq	Web interface
FlowSig (72)	Causal inference	scRNA-seq, spatial	Python

Abbreviations: PCOS, polycystic ovarian syndrome; scRNA, single-cell RNA.

## Conclusion

Clearly, our understanding of inflammation in PCOS requires significant refinement. This review challenges the long-held notion of “chronic inflammation” as an accurate descriptor for the immune status in PCOS. Instead, the current literature suggests tissue-specific immune mechanisms in PCOS, with distinct immune profiles observed across various organs involved in the disease process. The inconsistent findings from systemic inflammatory markers underscore the importance of shifting focus toward tissue-specific immune responses. Evidence from both human studies and animal models demonstrates unique immune alterations in tissues such as the ovaries, endometrium, and adipose tissue, which cannot be fully captured by analyzing circulating cytokines alone.

Moving forward, it is imperative that the field adopts a more nuanced approach to investigating immune-endocrine interactions in PCOS. Specifically, future research should prioritize the examination of cell-to-cell communication networks within affected tissues. This approach, facilitated by advanced single-cell technologies and computational tools, will be critical in establishing causal relationships between immune dysfunction and PCOS symptoms. Only through a comprehensive understanding of tissue-specific immune-endocrine interactions can we hope to develop successful immune-based therapies for PCOS. This shift in perspective from systemic inflammation to localized immune responses represents a critical step toward unraveling the complex pathophysiology of PCOS and identifying targeted treatment strategies for this prevalent endocrine disorder.

## Search Strategy and Study Selection

We conducted an exhaustive literature search focusing on tissue-specific inflammation in PCOS published within the last 5 years (2020-2025), with exceptions for earlier publications for historical context or lack of literature. The primary database searched was PubMed. Key search terms included combinations of “*PCOS*,” “*polycystic ovary syndrome*,” “*inflammation*,” “*immune*,” “*tissue-specific*,” and names of specific tissues (eg, “*ovary*,” “*adipose*,” “*liver*”). For systemic inflammation in PCOS, we prioritized recent meta-analyses to provide a comprehensive overview of current understanding. We manually reviewed reference lists of selected articles to identify additional relevant studies. The final selection of studies was based on their relevance to tissue-specific immune responses in PCOS and the quality of the research methodology.

## Data Availability

Data sharing is not applicable to this article as no datasets were generated or analyzed during the current study.

## Abbreviations

CRP: C-reactive protein
DHT: dihydrotestosterone
FSH: follicle-stimulating hormone
GnRH: gonadotrophin-releasing hormone
HPG: hypothalamic-pituitary-gonadal
IFN: interferon
IL-: interleukin
LH: luteinizing hormone
LPS: lipopolysaccharide
NFκB: nuclear factor κB
PCOS: polycystic ovarian syndrome
TLR4: toll-like receptor 4
TNF-α: tumor necrosis factor alpha
